# Editorial: Insights into RNA networks and biology

**DOI:** 10.3389/fmolb.2023.1156841

**Published:** 2023-02-17

**Authors:** André P. Gerber, Gian Gaetano Tartaglia

**Affiliations:** ^1^ Department Microbial Sciences, Faculty of Health and Medical Sciences, University of Surrey, Guildford, United Kingdom; ^2^ Center for Human Technologies (CHT), Istituto Italiano di Tecnologia (IIT), Genova, Italy

**Keywords:** RNA, modelling, biochemistry, high-throughput sequencing, metabolism, translation

In our Research Topic “Insights into RNA Networks and Biology” we gathered a number of articles that relate to the newest developments in the RNA field. Starting from computational works (Pepe et al**.** and Vandelli et al.) and passing through network analysis (Yang et al. and Zhang et al.), we present approaches for biochemical characterization of protein-RNA interactions (Giambruno et al. and Kieffer et al.)**,** implication of long non-coding RNAs (lncRNAs) in disease (Fan et al. and Li et al.) and investigation of the role of RNA molecules and translation in physiology (Buchanan et al. and Zhang et al.).

Several theoretical methods for studying physical interactions between RNA and proteins are currently being developed. The review by Pepe et al. summarizes the most recent advances in the algorithms, especially to predict circular RNA (circRNA) and long non-coding RNA (lncRNA) interactions with RNA-binding proteins (RBPs). The authors emphasize the need for developing larger interaction datasets to increase performance and expand on approaches amenable to machine learning. In their Perspective, Vandelli et al. provide an example on how the integration of RNA-protein interaction data with computational analysis tools can lead to new hypotheses. The authors used data from four experimental and one computational study of interactors of SARS-CoV-2 genomic RNA. Although hundreds of proteins have been identified across the different studies, only twenty-one appear in all the experiments to interact with SARS-CoV-2 RNA. The identified proteins refer to known or predicted stress granule forming proteins, which leads to the intriguing hypothesis that a mechanism of action for SARS-CoV 2 could be to target proteins that attract other partners through phase separation.

Two articles focus on transcriptome analysis for construction of RNA-RNA networks. Yang et al. performed a systematic analysis of mRNA, microRNA (miRNA) and circRNA expression in type II alveolar epithelial cells (ATII) in Tibetan pigs. These pigs live at high altitude and developed an efficient metabolism for living at lower oxygen levels than “standard” landrace pigs. The authors simulated a hypoxic environment for cultured ATII cells of tibetan pigs and landrace pigs grown under normoxic and hypoxic conditions. The differential expression of circRNA, miRNAs and mRNAs were combined to construct competing endogenous RNA (ceRNA) networks for these cells, thereby identifying critical regulatory modes for metabolic control. A similar approach was used by Zhang et al. that explored the transcriptomic network of Rotator cuff tears (RCT), the most common cause of shoulder dysfunction. Specifically, the authors performed RNA-seq analysis of five patient samples with RTC in supraspinatus muscles and matched unharmed subscapularis muscles from the same individual to dissect the dysregulated transcriptome including mRNAs, miRNAs, lncRNAs, and circRNAs. Based on network construction considering the ceRNA hypothesis, they suggested dysregulation of the ceRNA network in RTC through several ncRNAs. Hence, the authors propose that these ncRNAs could play a role in the development of RCT, possibly adding a new angle for therapeutic approaches.

Cross-linking is often used to preserve functional protein-RNA interactions present in the cell. The review by Giambruno et al. describes recently developed complementary proximity labeling techniques to map RNA-protein interactions in mammalian cells. This technology permits the identification of relatively transient interactions as well as poorly expressed molecules by relying on the inducible activity of enzymes (biotin ligases or peroxidases) that are expressed in living cells and biotinylate amino acids and nucleic acids of binding factors in proximity–within 20 nM range. Besides the interaction with their target RNAs, RBPs often interact with other proteins which further influence the fate of ribonucleoprotein (RNP) complexes. Kieffer et al. combined affinity purification and mass spectrometry (MS) to define the network of nuclear proteins interacting with the N-terminal region of FMRP. FMRP is encoded by the *FMR1* gene (Fragile X messenger ribonucleoprotein 1) whose absence of expression results in Fragile X-syndrome (FXS), the most common inherited form of intellectual disability. While it was well known that FMRP regulates translation of subsets of mRNA in the cytoplasm, the protein shuttles between the nucleus and cytoplasm but little was known about its nuclear functions. Hence, this study adds a base to further mechanistic studies on its nuclear functions in neuronal physiology.

Two review articles highlight the specific and broader implications of lncRNAs in disease. Fan et al. discuss the role of lncRNAs and aerobic glycolysis in tumorigenesis and tumor progression and explore the interaction networks to provide insights into therapeutic targets for treatment. The authors suggest that lncRNAs could regulate key enzymes related to glycolysis to promote aerobic glycolysis (examples include *PKM2, LDH, HK2* and *GLUT*). An important highlight of the work is that lncRNAs can act as sponges for miRNAs to regulate expression of enzymes or modulate signaling pathways. The association of lncRNAs with enzymes could also promote formation of protein complexes (examples include lncRNA *HULC* binding to LDHA, PKM2) thus conferring additional functions. The article by Li et al. elaborates on the relationship between lncRNAs and diseases. In the case of tumor development, the authors report that a lncRNA can act either as proto-oncogene or tumor suppressor. As for neurodegenerative and psychiatric diseases, examples are provided for *DISC1* (schizophrenia); *PINK1* (endocrine diseases), *MALAT-1* and *NEAT1/2* and others. One important case is *BACE1* antisense (*BACE1AS*) that forms complex with *BACE1* messenger RNA to increase the stability of the latter thus promoting aggregation of β-amyloid protein in Alzheimer’s disease.

Besides the many ncRNA and viral RNAs concerned in this Research Topic, two articles focus on mRNAs for cell fate determination and in translation. In their perspective, Buchanan et al. discuss the potential role of ribosome heterogeneity in brain plasticity. After briefly reviewing the importance of translation regulation in neuroplasticity, the authors highlight recent results showing that complex brain functions such as sleep and learning have a significant impact on ribosomal protein (RP) expression. As sleep is critical in promoting brain plasticity, the authors then propose that changes in RP stoichiometry during sleep could establish a mechanism for regulation of translation of subsets of mRNAs that code for proteins mediating brain plasticity. Zhang et al.investigate the regulation of Hsp70 chaperones *Ophraella communa.* In concert with many cofactors, Hsp mediates essential activities such as protein folding and assembly. Normally Hsp70 is located in the cytoplasm, however, when cells are stimulated by heat stress, the protein is transferred to the nucleus. In the case of *O. communa*, an important biological control agent of the ragweed *Ambrosia artemisiifolia* worldwide, Hsp70 was found to be highly expressed in the female ovaries and male testes and induced by mating. The regulatory mechanisms of Hsp70 and the investigation of its RNA stability and translation should be further investigated.

